# Evaluating Deep Learning models for predicting ALK-5 inhibition

**DOI:** 10.1371/journal.pone.0246126

**Published:** 2021-01-28

**Authors:** Gabriel Z. Espinoza, Rafaela M. Angelo, Patricia R. Oliveira, Kathia M. Honorio

**Affiliations:** 1 School of Arts, Sciences and Humanities, University of Sao Paulo, Sao Paulo, Sao Paulo, Brazil; 2 Federal University of ABC, Santo Andre, Sao Paulo, Brazil; University of Craiova, ROMANIA

## Abstract

Computational methods have been widely used in drug design. The recent developments in machine learning techniques and the ever-growing chemical and biological databases are fertile ground for discoveries in this area. In this study, we evaluated the performance of Deep Learning models in comparison to Random Forest, and Support Vector Regression for predicting the biological activity (pIC_50_) of ALK-5 inhibitors as candidates to treat cancer. The generalization power of the models was assessed by internal and external validation procedures. A deep neural network model obtained the best performance in this comparative study, achieving a coefficient of determination of 0.658 on the external validation set with mean square error and mean absolute error of 0.373 and 0.450, respectively. Additionally, the relevance of the chemical descriptors for the prediction of biological activity was estimated using Permutation Importance. We can conclude that the forecast model obtained by the deep neural network is suitable for the problem and can be employed to predict the biological activity of new ALK-5 inhibitors.

## Introduction

Cancer is considered one of the leading causes of death in the world and can be defined as a disease that arises from cumulative changes in the genetic material of normal cells, which change until they become malignant [1]. Cancer is related to a collection of more than 100 types of different diseases that share the disordered growth of abnormal cells with invasive potential. Its origin may occur due to multifactorial conditions, where these causal factors may act together or in sequence to initiate or promote cancer (carcinogenesis). Although it seems to be a current disease, cancer is known from the earliest human societies that have recorded their symptoms [2, 3].

The corrected annual global calculation for the sub-record points to 640,000 new cancer cases [4]. Since cancer incidence rates have substantially increased in last decades, several research groups are studying ways to treat its various forms, leading to the discovery and studies of several biological targets related to this pathology [5, 6]. Among these many targets, there is great interest in the TGF-β type I receptor (type I receptor transforming growth factor-beta), which is also known as ALK-5 (activin receptor type-5 kinase, or receptor-like activin kinase 5), member of the TGF-β superfamily. The TGF-β growth factor superfamily is essential in maintaining homeostasis, as well as in inducing wound healing, controlling the immune system. It is also linked to various other biological processes such as alveolarization, immune cell recruitment, platelet aggregation, apoptosis, and proliferation. However, in a cancer-related scenario, TGF-β presents an opposing role, promoting growth, invasion, and metastasis [7]. Particularly, it has been shown that ALK-5, the main mediator of TGF-β signaling, is paradoxically a potent tumor suppressor in normal cells but a growth and metastasis enhancer in late-stage cancer [8]. In spite of the fact that such achievement makes the window for targeting ALK-5 small, many studies have been carried out on this target due to its clinical relevance. For instance, Yue et al. [9] achieved promising results by using galunisertib, an ALK-5 inhibitor, for treating two types of myelofibrosis, a bone marrow blood cancer, in mouse models.

In this scenario, many approaches, such as molecular modeling and medicinal chemistry tools, can be applied to investigate and analyze the interaction of drug candidates with the ALK-5 receptor. More specifically, drug design can be streamlined and helped from quantitative studies that modeling the relationships between chemical structure of substances and their target’s biological activity. In this sense, drug design is a complex task that involves interpreting the mechanism of action of a certain compound and its interaction with the biological target.

Due the progressive improvement in computational resources and the increasing amount of publicly available data in repositories such as PubChem [10] and ChEMBL [11, 12], machine learning (ML) methods have been widely used for identifying potential drugs [13]. Some examples of ML techniques successfully applied in the drug discovery context are support vector machines (SVM), k-nearest neighbors (kNN), naïve Bayes, and decision trees [14].

Quantitative structure-activity relationship (QSAR) is a method largely used for predicting the activity of a substance against a biological target through the relationships between biological data and molecular descriptors that are dependent on the molecular structure [15]. To model these relationships, different ML techniques can be employed, such as Random Forests (RF) and SVM [16]. The predictions obtained by the ML models can be used to minimize the amount of laboratory experimentation required to discover new bioactive molecules.

More recently, applications involving Deep Learning models have gained attention due to their ability to extract important features from raw data and handle complex tasks [17], such as drug design. For instance, the Focused Library Generator designed by Xia et al. [18] was able to design new inhibitor molecules with desired properties from scratch. Stokes et al. [19] implemented Deep Learning for antibiotic prediction, which led to the discovery of a structurally distant antibacterial molecule. Zhavoronkov et al. [20] developed a deep generative model to design small molecules, finding potent inhibitors of discoidin domain receptor 1, a target related to fibrosis, among other diseases. Other recent work refers to druGAN [21], a deep adversarial generative autoenconder employed in *de novo* design of drugs with desired properties.

Deep Learning can also be used to predict binding affinity between a ligand and a biological target, another imperative information in the drug discovery and design processes. For example, *K*_*DEEP*_ [22] uses deep 3D-convolutional neural networks to estimate the binding affinity, making such information easier to predict and therefore facilitating chemistry pipelines. In addition, BindScope [23], another deep 3D-convolutional network, was proposed to discriminate between active and inactive compounds.

Many machine learning computational libraries have become available for biological and chemical researches in recent years. The present study used TensorFlow [24, 25], an open-source software library for machine learning developed by Google and Keras [26], to implement a DNN model in order to predict the biological activity (inhibition) of a given molecule against the ALK-5 receptor, which could be used to identify drug candidates for cancer treatment through virtual screening (VS).

## Material and methods

**Fig 1:**
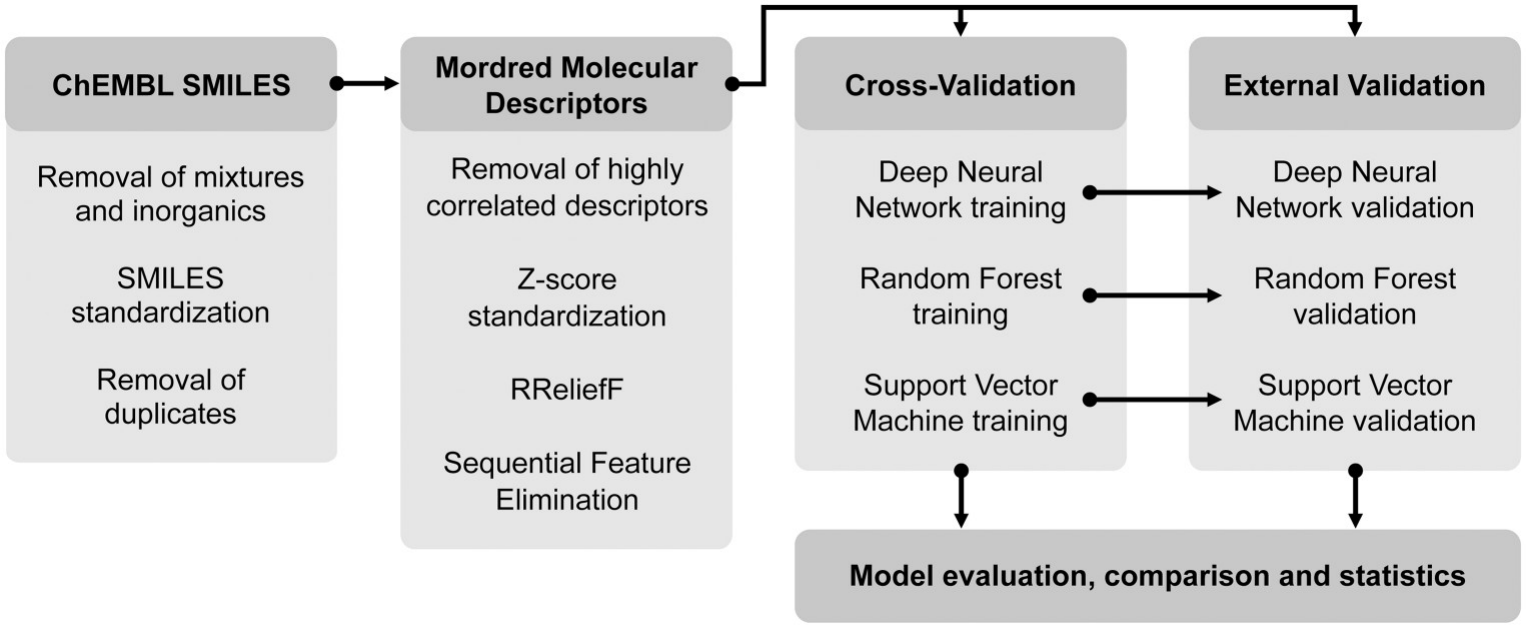
Workflow for the methods used in this work to curate data and train regression models.

This work aims at generating and comparing the performance of three different machine learning regression models for predicting IC_50_ (half-maximal (50%) inhibitory concentration) values based on data for several compounds with known biological activity against ALK-5. The workflow used in this process is shown in Fig 1.

### Data collection

Compounds with known activity against the TGF-beta receptor type I were obtained from the ChEMBL database (target ID CHEMBL4439), resulting in 1317 compounds with experimentally measured IC_50_. However, only substances with accurate measurements achieved from cellular and protein binding assays were retained in our analyses, reducing the dataset to 859 molecules.

Further data curation was done based on the recommendations from Fourches, Muratov, and Tropsha [27], which consist in removing inorganic compounds and mixtures of substances. The SMILES notations provided by ChEMBL were converted to their universal canonical forms and all hydrogens were considered implicit using Open Babel [28]. It is important to mention that RDKit automatically converts the internal molecular representations to feed the right information into each descriptor calculation. Therefore, this standardization was only made externally to ensure all SMILES were valid and that RDKit would be able to interpret them. Finally, duplicates were removed. If duplicates had pIC_50_ values within a 0.1 margin, the average value was kept, otherwise, all were discarded. After this process, the resulting and final dataset had 558 unique molecules.

**Fig 2:**
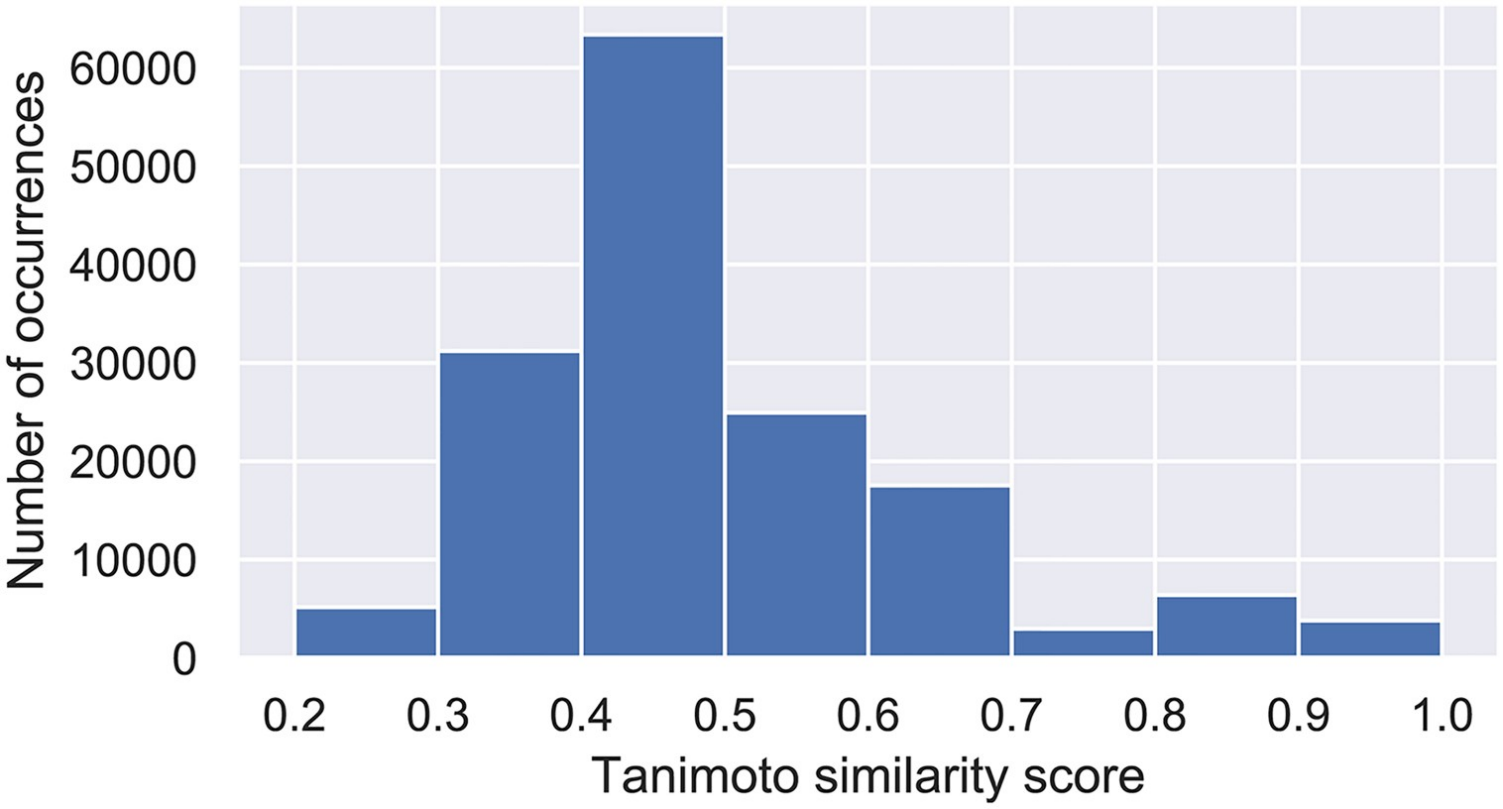
Histogram of Tanimoto similarity scores between all possible unique pairs for the 558 molecules in the dataset. These values were calculated by the RDKit Python library using 2048 bits daylight-like fingerprints (the similarity score averaged at 0.493).

The Tanimoto similarity scores for all pairs of molecules and their general distribution are shown in Fig 2. The fingerprints needed for the similarity score calculation were obtained using the RDKit Python library and 2048 bits topological fingerprints were employed in this analysis. It is interesting to note that the biological activity values of the compounds ranged from 0.57 nM to 99000 nM. In order to improve the numerical stability across all models, these values of IC_50_ were converted to pIC_50_ (−*log IC*_50_).

### Cross-validation

The steps including feature selection, parameter tuning, and model training were carried out using 5-fold cross-validation on 2/3 of the entire dataset (n = 372). All performance metrics calculated in such experiment considered only predictions made for the test sets (not seen during the training phase). Further, additional results were obtained by testing the models on the remaining 1/3 of the entire dataset (n = 186), referred to as validation set. It is important to mention that all experiments used the same split of data in order to make the results comparable.

### Calculation and selection of chemical descriptors

Chemical descriptors, defined as the result of a mathematical procedure that transforms the encoded chemical information of a molecule into a useful number or the result of some standardized experiments, were calculated using the Mordred Python library [29]. Mordred calculates over 1800 standard molecular descriptors, including all implemented by RDKit (seven modules) and original implementations (42 modules). The number of standard descriptors calculated by Mordred is comparable to other widely used software, so it can be used as an alternative to calculate descriptors for QSAR studies.

Although Mordred generated a considerable amount of descriptors, many of them were redundant. More specifically, some features with the same meaning may appear repeatedly on different types of descriptors. There were also descriptors whose values never change, bringing no relevant information to the models. Considering this scenario, we can observe that selecting descriptors is an essential step in the modeling process. To find the most relevant descriptors and identify a suitable number of features, highly correlated descriptors were initially discarded (R^2^ > 0.95), resulting in 856 variables. To improve the numerical stability, all resulting descriptors were then standardized by z-score, i.e. they were centered and scaled to achieve zero mean and unit variance. RRefliefF [30], a filter method that scores features based on the differences on feature values between nearest-neighbor instance pairs, was then applied. The 50 highest-scoring features were initially selected for a grid search process in order to find optimal parameters of a deep neural network architecture.

After that preliminary step, a wrapper method known as Sequential Backward Floating Selection (SBFS) [31] was then further applied using to the tuned deep neural network architecture, using the Mean Squared Error as a score function. The selection process consisted in removing one feature at a time based on the regression performance until only one feature is left. This algorithm can also include features at each step if the increase in performance (in this case, decrease in Mean Squared Error) is greater than removing any other feature. These feature selection steps only considered the dataset portion obtained for the 5-fold cross-validation procedure, as previously described.

### Principal component analysis

Once the most relevant descriptors to the neural network model were selected, further study about them was conducted, aiming at better understanding their characteristics and possible relations to the biological activity responsible for the ALK-5 inhibition. In this sense, we performed a detailed analysis on the chemical properties from using Principal Component Analysis (PCA) [32].

### Neural networks for Deep Learning

An artificial neural network (ANN) architecture consists in interconnected layers of many neurons (also commonly referred to as units or nodes) that attempts to mimic the natural behavior of the nervous system. In this work, we consider a feedforward architecture, where all neurons between two consecutive layers are fully connected and the information flows only in one direction, from the input to the output units.

Even though the Stochastic Gradient Descent algorithm has been widely used for the training process due to its accuracy; the Adam algorithm [33] has been gaining popularity for its speed and consistent performance. It is a first-order gradient stochastic optimization method well suited to applications involving large datasets and high-dimensional parameter spaces. Such algorithm has some notable advantages in comparison to other methods, such as the invariance of parameter magnitude to gradient rescaling, good performance on noisy and sparse gradients, and low memory requirements. Adam achieves these benefits by using estimates of first and second moments of the gradients in order to compute individual adaptive learning rates for different parameters.

To reduce convergence time and prevent overfitting, as well as the vanishing gradient problem [34], the rectified linear activation function (ReLU) was utilized as the activation function [35]. Early Stopping was also adopted since it is a method that ends the training phase if there is no improvement in performance over a certain number of epochs (in this case, 100 epochs).

Another recent development in this area is known as Dropout [36], which is a regularization technique that randomly and temporarily removes a fixed proportion of different neurons and their respective connections from the network in each training step. Such strategy is useful for avoiding complex co-adaptations on training data, therefore reducing overfitting.

Considering the advantages of the approaches previously discussed, a ReLU-based DNN with Dropout and Early Stopping was adopted in this study, which is usually enough to prevent overfitting and the vanishing gradient problem [37]. The initial weights for the network were chosen by the method proposed by Glorot et al. [35], in which the model convergence is faster and more consistent. This method works by initializing the weights of a layer with values from a normal distribution with zero mean and variance inversely proportional to the number of neurons associated with a single weight. The optimum architecture, including the dropout rate, was obtained by performing a grid search, a method of determining the best combination of parameters by extensively training models on all possible combination within a given configuration set. In this work, we used the Scikit-learn [38] implementation for this task.

### Other machine learning methods

In order to assess the deep neural network performance, two other machine learning models (a Random Forest regressor [39, 40], and a Support Vector Machine regressor [41]) were trained on the same dataset. Both models were implemented using the Scikit-learn library.

Random Forest (RF) is an ensemble composed of several decision trees, where each of them uses a random subset of instances in the dataset and a final prediction result is obtained by consensus (typically as the mean prediction for all trees). RF is especially useful as it naturally handles correlations and presents a lower sensitivity to hyperparameter modifications when compared to other methods. On the other hand, support vector machines for regression (SVR) perform high-dimensional mapping by using nonlinear functions to linearly estimate an unknown regression value. The optimal parameters for both methods were found using grid search.

### Metrics

The coefficient of determination (*R*^2^) is used in this study to evaluate the model performance by comparing the predicted activities ${(\hat{y}}_{i})$ with the observed values (*y*_*i*_) available in the test set. *R*^2^ assesses the concordance between these values when compared to the simple average $\left(\overset{-}{y}\right)$ of the observed data and it can be calculated as:

$$R^{2}=1-\frac{\sum_{i}^{n}{\left(y_{i}-{\hat{y}}_{i}\right)}^{2}}{\sum_{i}^{n}{\left(y_{i}-\overset{-}{y}\right)}^{2}}$$ (1)

Mean squared error (MSE) and mean absolute error (MAE) are popular metrics to evaluate regression models and they were also used in this study (see Eqs 2 and 3).

$$MSE=\frac{1}{n}\sum_{i}^{n}{\left(y_{i}-{\hat{y}}_{i}\right)}^{2}$$ (2)

$$MAE=\frac{1}{n}\sum_{i}^{n}\left|y_{i}-{\hat{y}}_{i}\right|$$ (3)

### Hardware and training environment

The hardware used in this study consists of one Nvidia Tesla T4 GPU card (320 Turing Tensor cores, 2560 CUDA cores and 16GB of GDDR6 VRAM), one single-core Intel Xeon Processor E5-2699 v3 (2.3Ghz and 45MB Cache) and 16GB of DDR4 RAM. The DNN model was trained using the GPU implementation of TensorFlow while all other processes were performed on the CPU mode.

The Python programming language was used for the present study. Along with it, some libraries were also employed: TensorFlow [24], Keras [26], NumPy [42], Pandas [43], Scikit-learn [38], Matplotlib [44], RDKit [45], and Mordred [29].

## Results and discussion

### Parameter tuning

**Fig 3:**
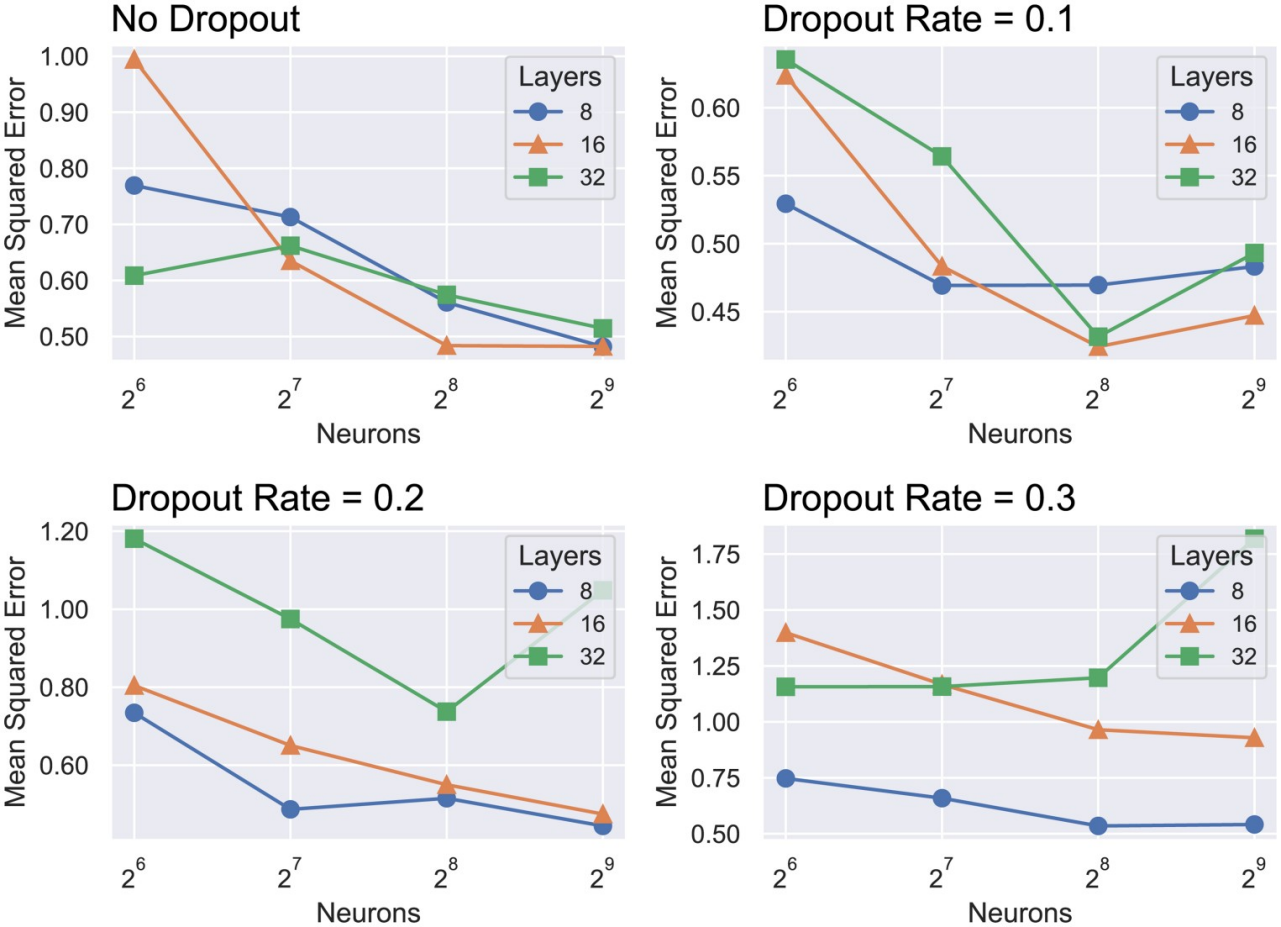
Summary of the grid search process for choosing the hyperparameters for the Deep Neural Network.

The best Deep Neural Network architecture was found to have the following parameter values: a dropout rate of 10% and 16 hidden layers with 256 units each. The grid search method is summarized in Fig 3. The objective function considered in all scenarios was the Mean Squared Error.

**Fig 4:**
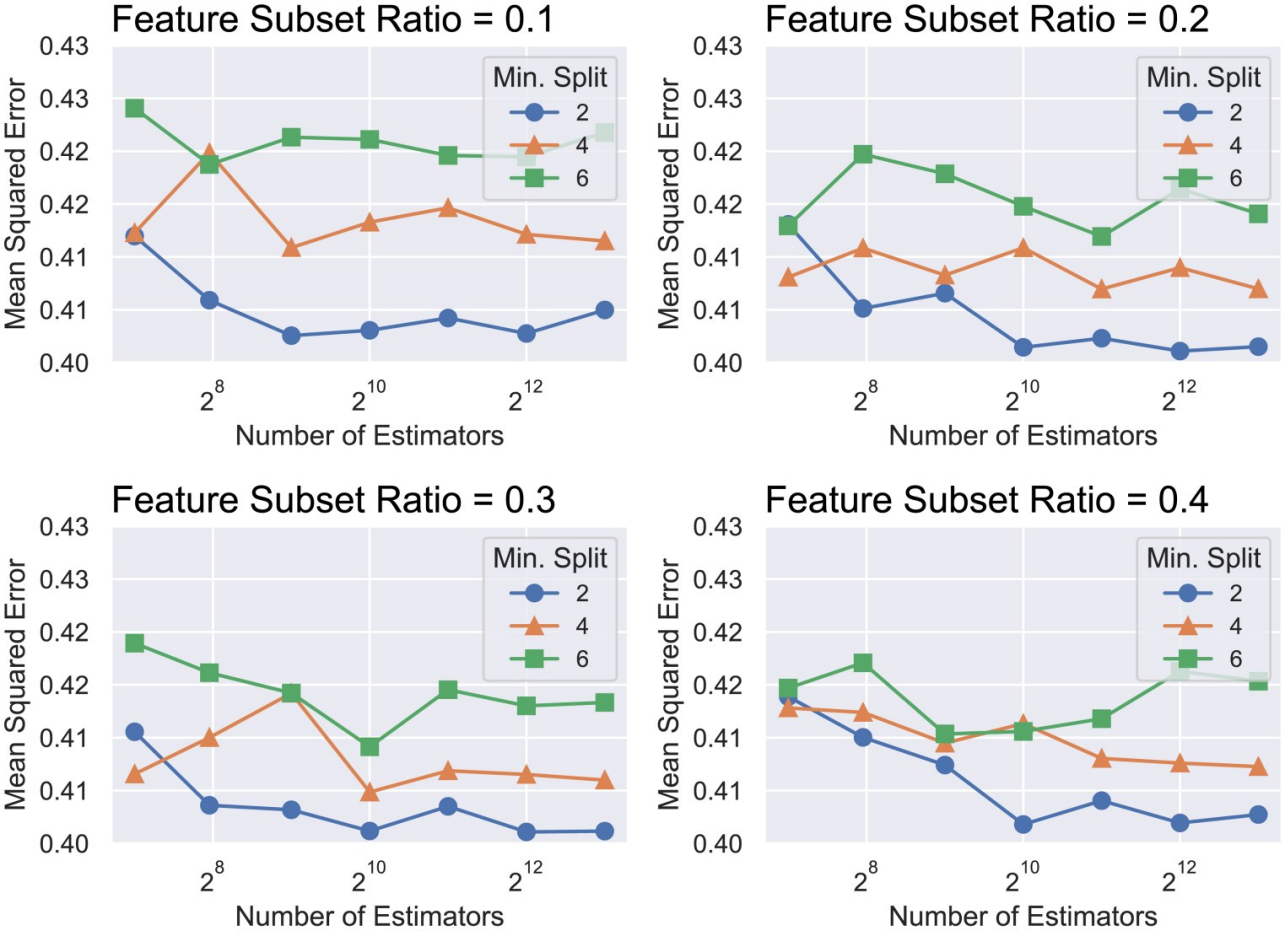
Summary of the grid search process for choosing the hyperparameters for the RF model.

The best RF model obtained with grid search consists of 4096 trees with a minimum split size of 2 (so all possible splits are performed) and a subset size of 30% of the number of selected descriptors, as presented in Fig 4.

**Fig 5:**
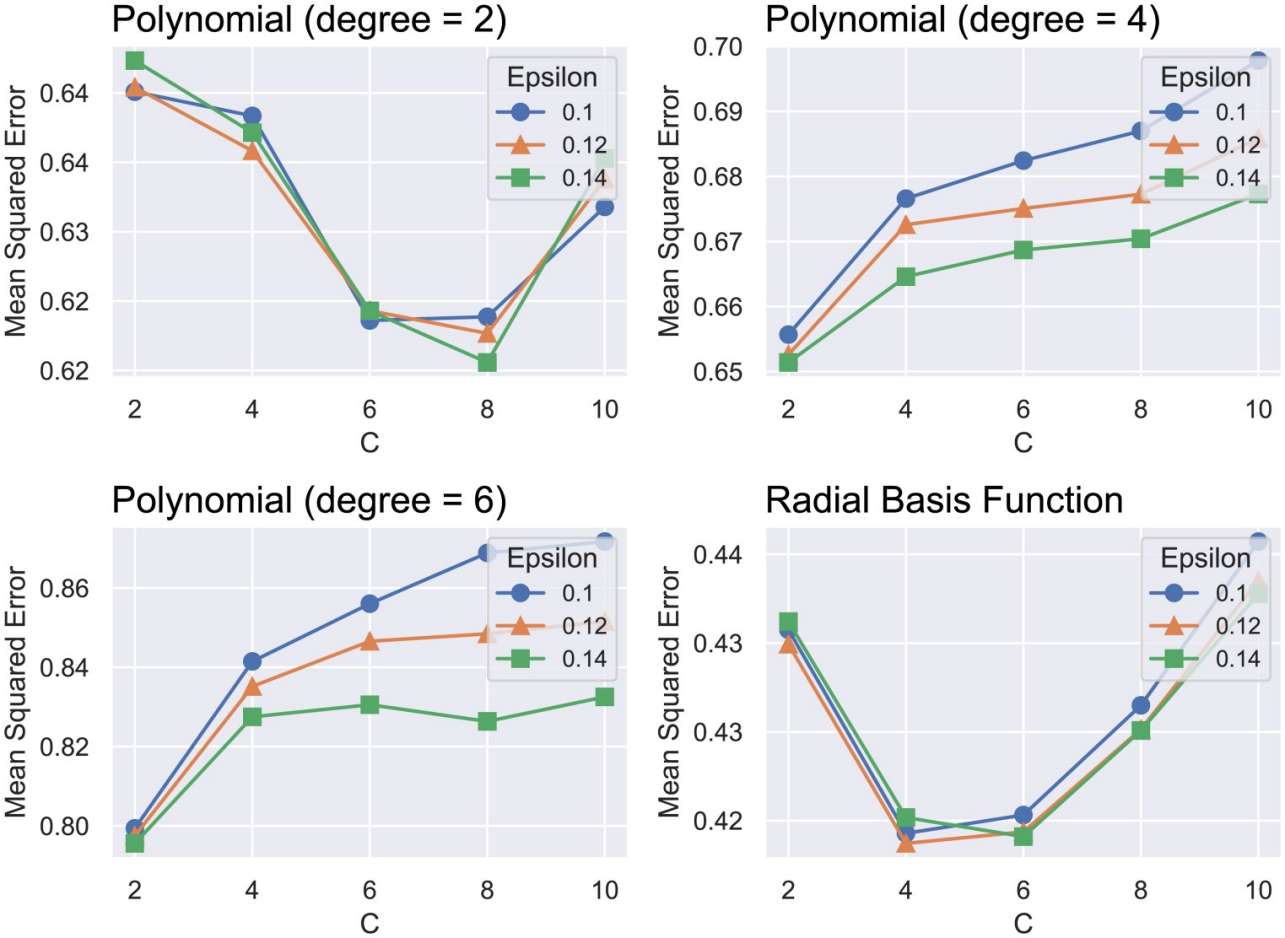
Summary of the grid search process used to select the hyperparameters for the SVR model.

The best configuration for the SVR model obtained by using grid search is consisted by a radial basis function kernel, a penalty parameter C of 4 and an epsilon of 0.12, as displayed in Fig 5. Note that, for all three models, the grid search was performed on the 5-fold cross-validation section of the dataset that corresponds to 2/3 of the entire dataset.

### Feature selection

**Fig 6:**
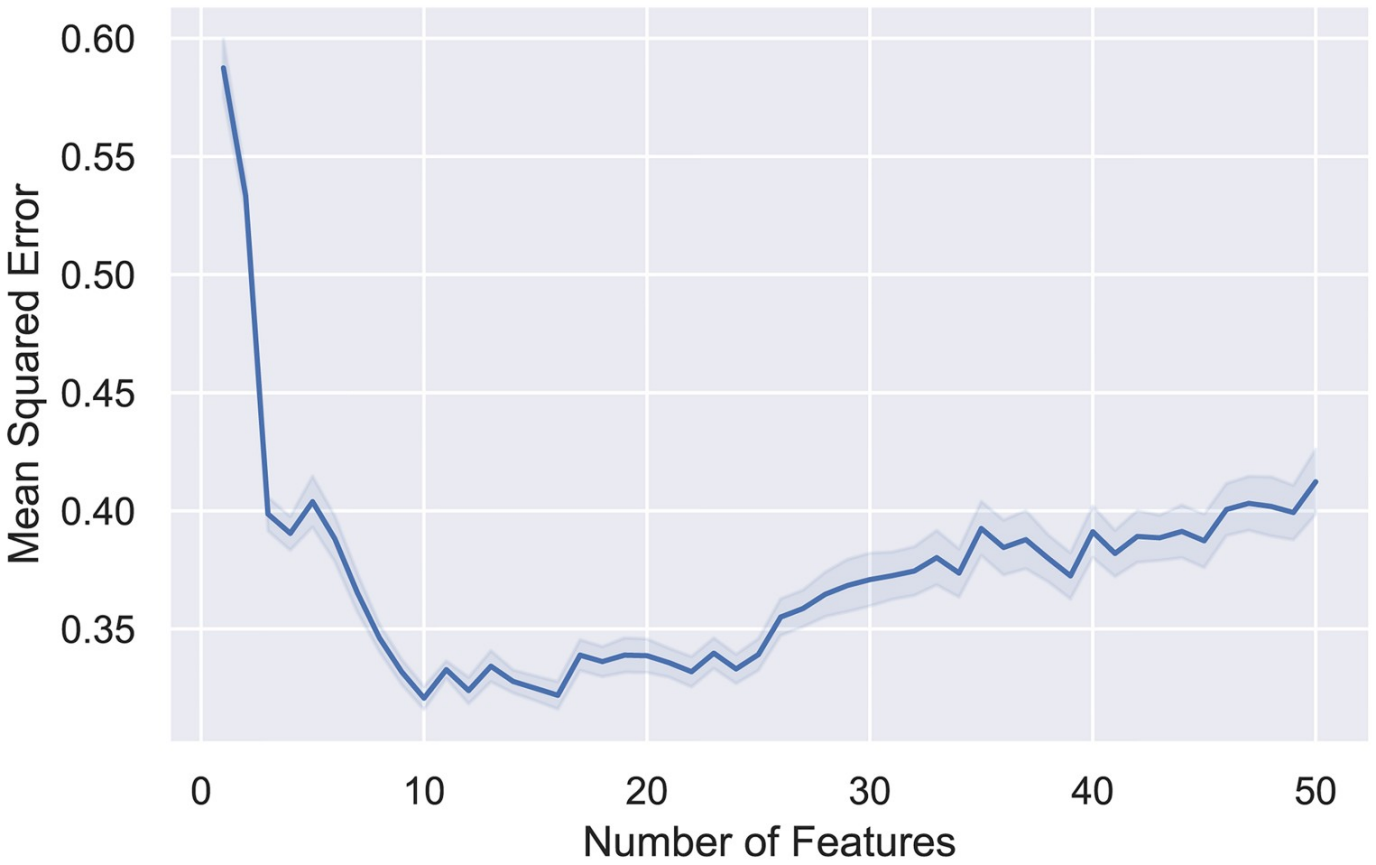
MSE values obtained for the models trained on different numbers of features following Sequential Backward Floating Selection with a 95% confidence interval.

The results obtained after inputting the 50 best descriptors based on RReliefF into the Sequential Backward Floating Selection process are displayed in Fig 6, which consisted in 10 descriptors selected for training the ML models according to the lowest MSE values.

### Cross-validation

In order to evaluate the performance of the DNN model in comparison to the other ML methods, we trained all of them on the same dataset experimental configuration so that to make their results can be comparable. This comparison encompasses measures calculated on test sets in the cross-validation procedure and on the validation (external) set. These results are summarized in Table 1, in which MAE, MSE, and R^2^ are presented.

**Table 1:** Performance measures for the ML models calculated on test and validation sets.

Metric	DNN	RF	SVR
Test R^2^	0.673	0.650	0.587
Test MAE	0.450 ± 0.042	0.477 ± 0.042	0.500 ± 0.048
Test MSE	0.373 ± 0.080	0.399 ± 0.068	0.471 ± 0.096
Validation R^2^	0.658	0.623	0.617
Validation MAE	0.480 ± 0.053	0.484 ± 0.059	0.490 ± 0.059
Validation MSE	0.366 ± 0.078	0.403 ± 0.092	0.409 ± 0.095

Coefficient of determination (R^2^), mean absolute error (MAE) and mean squared error (MSE) measured on test and validation sets with confidence intervals of 95%.

From Table 1, we can see that the deep neural network achieved the best performance (*R*^2^ = 0.673) when compared to the random forest regressor (*R*^2^ = 0.650) and the support vector regressor (*R*^2^ = 0.587) in the cross-validation section.

**Fig 7:**
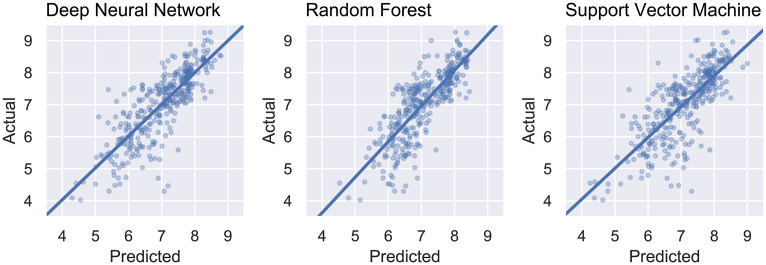
Scatter plots and regression lines obtained by the cross-validation procedure using DNN, RF, and SVR (actual versus predicted pIC_50_).

Fig 7 displays the plots of actual *versus* predicted values obtained from the DNN, RF, and SVR models. We can observed that the dispersion in the test predictions obtained by the neural network model has a better centered regression line when compared to the other methods. This implies that its predictions are better balanced across different values, being generally more consistent. The RF model seems particularly less reliable when predicting low pIC_50_ values, whereas the SVR model has visibly higher errors.

### Applicability domain

Since the data utilized to train and evaluate the models is limited in terms of chemical diversity, as shown in Fig 2, the definition of an applicability domain (AD) becomes an important step to ensure future predictions are reliable within expect errors. AD is defined as a region in the chemical space that comprises the molecules used to train and validate the models. It allows the inference of a prediction given how similar its corresponding molecule is to the data used to develop the model [46]. Therefore, the compounds can be assessed as outliers and this approach is an useful tool to define the space of interpolation of the models.

**Fig 8:**
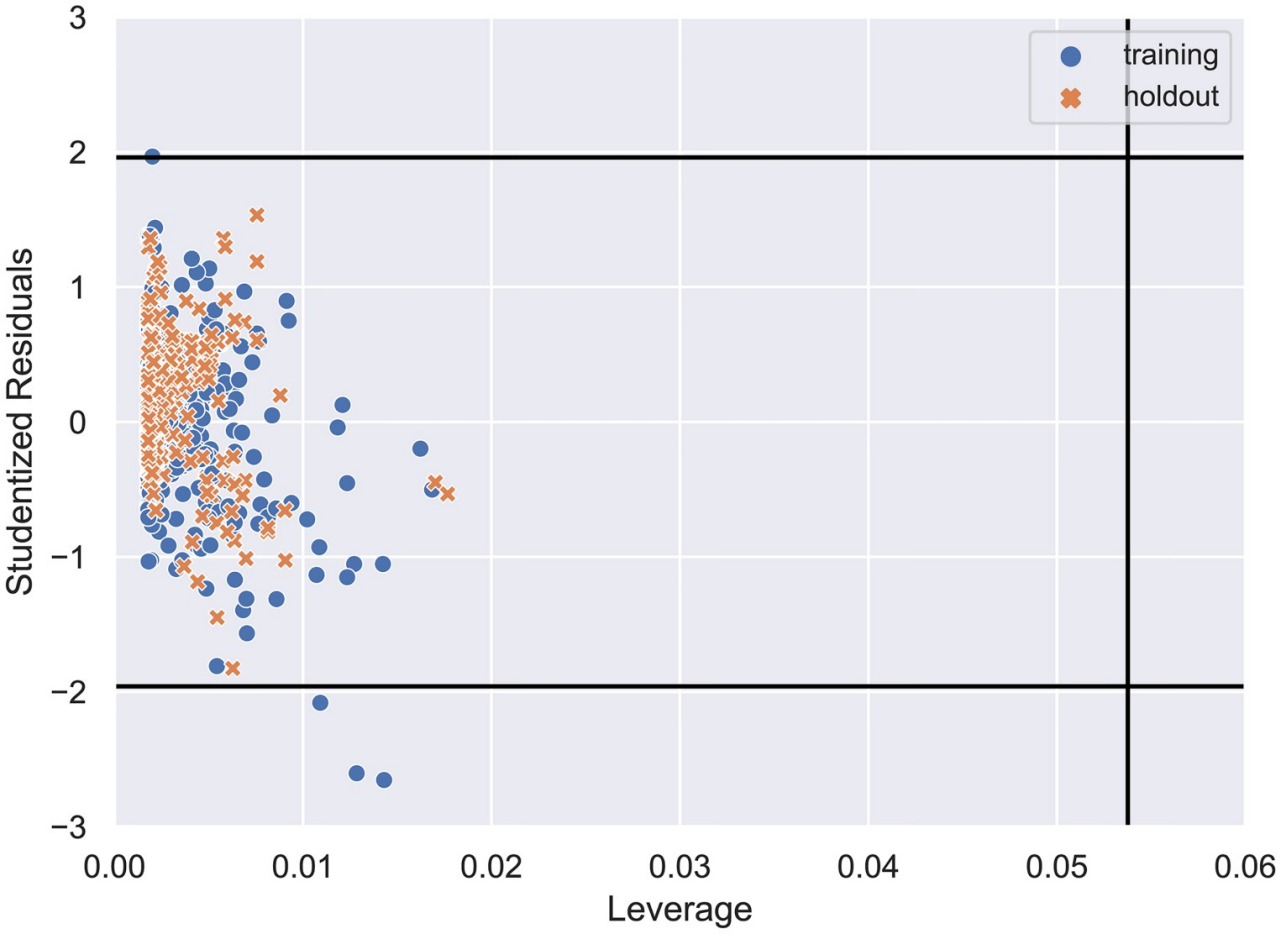
Studentized residuals vs. leverage values for the DNN regression. The horizontal lines represent a 95% probability level, and the vertical critical line is set to three times the number of latent variables (10) divided by the total number of samples (558).

One of the premises of the QSAR models suggests that compounds with similar structure could have similar biological activity [47]. So, we decided to obtain the applicability domain for the dataset used to construct the DNN model that is displayed in Fig 8.

From Fig 8, we can see that only three samples in the training set were outside the critical values for Studentized residuals, which represent a probability level of 95% assuming a normal distribution. OECD (Organization for Economic Cooperation and Development) has established some principles to validate QSAR analyses and one of them suggests that a model should only be employed within its applicability domain [48]. Therefore, the results obtained indicate that the model is valid within this domain and it can be used to predict the biological data of new samples that are within the limits in this domain.

### Analysis of descriptor weights on the regression models

**Fig 9:**
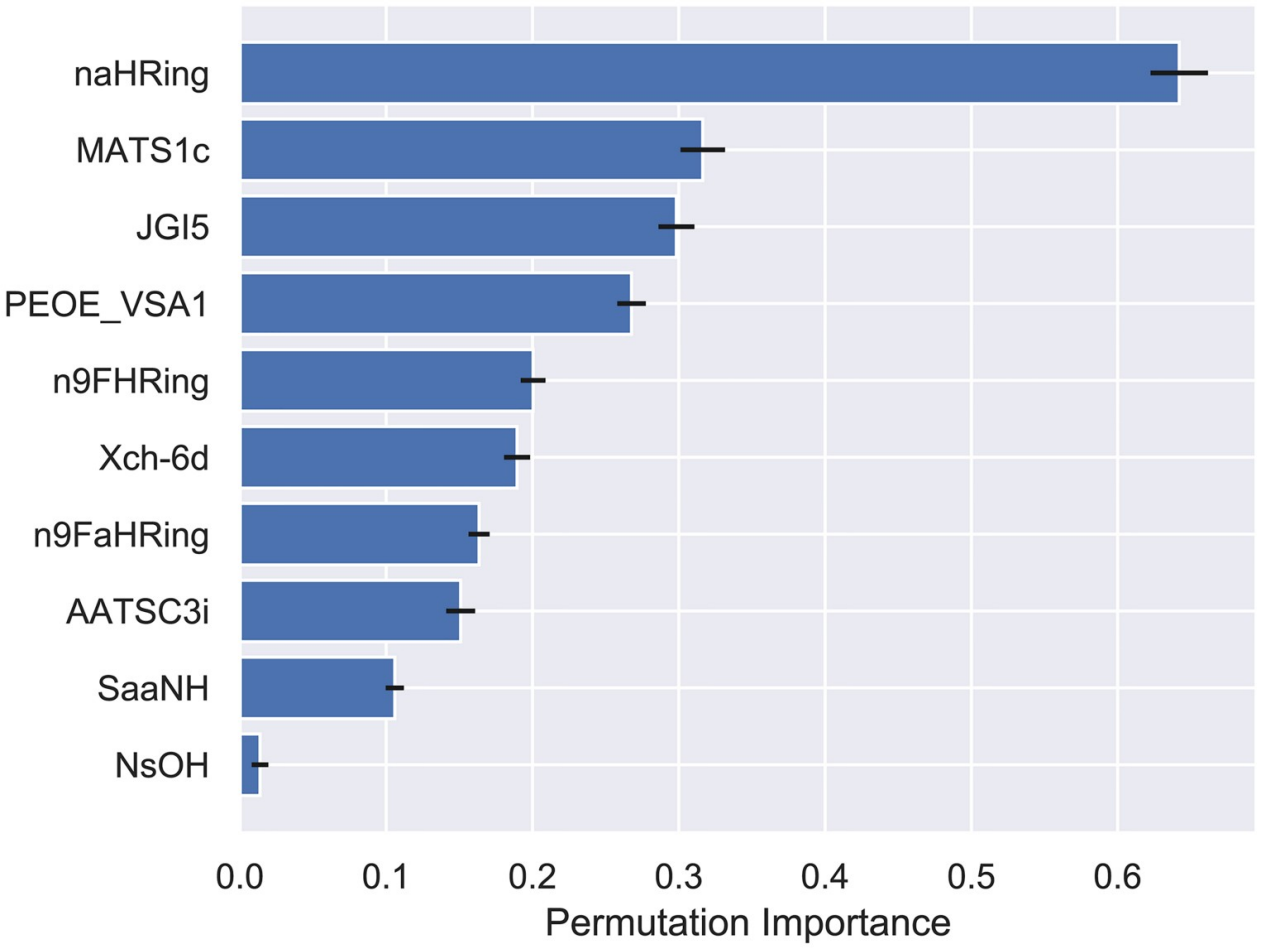
Permutation importance of descriptors in the DNN model, with 95% confidence interval.

Model interpretation and extraction of important features are crucial steps to understand how the model predictions are made and which of the hundreds of descriptors are the most relevant to comprehend the action mechanism of the compounds under evaluation. To accomplish that, the previously discussed feature selection method was implemented. The selected descriptors and their permutation importance in the DNN model can be seen in Fig 9. The permutation importance estimates the dependence of the model measuring how much the output of any regressor changes when all values of a single feature are shuffled. This process was repeated 1000 times for each of the 10 features.

The 10 selected descriptors are originated from six descriptor groups according to the Mordred modules: ring count, autocorrelation, topological charge, MOE-type, Chi connectivity and electrotopological state. The most important descriptor, *naHRing*, is simply the number of aromatic rings containing heteroatoms. *MATS1c*, on the other hand, is an autocorrelation descriptor that encodes both molecular structure and physicochemical properties. *JGI5* is 5-ordered mean topological charge. In sum, the most relevant descriptors indicate that structural, electronic and physicochemical features are essential to compounds have biological activity.

**Fig 10:**
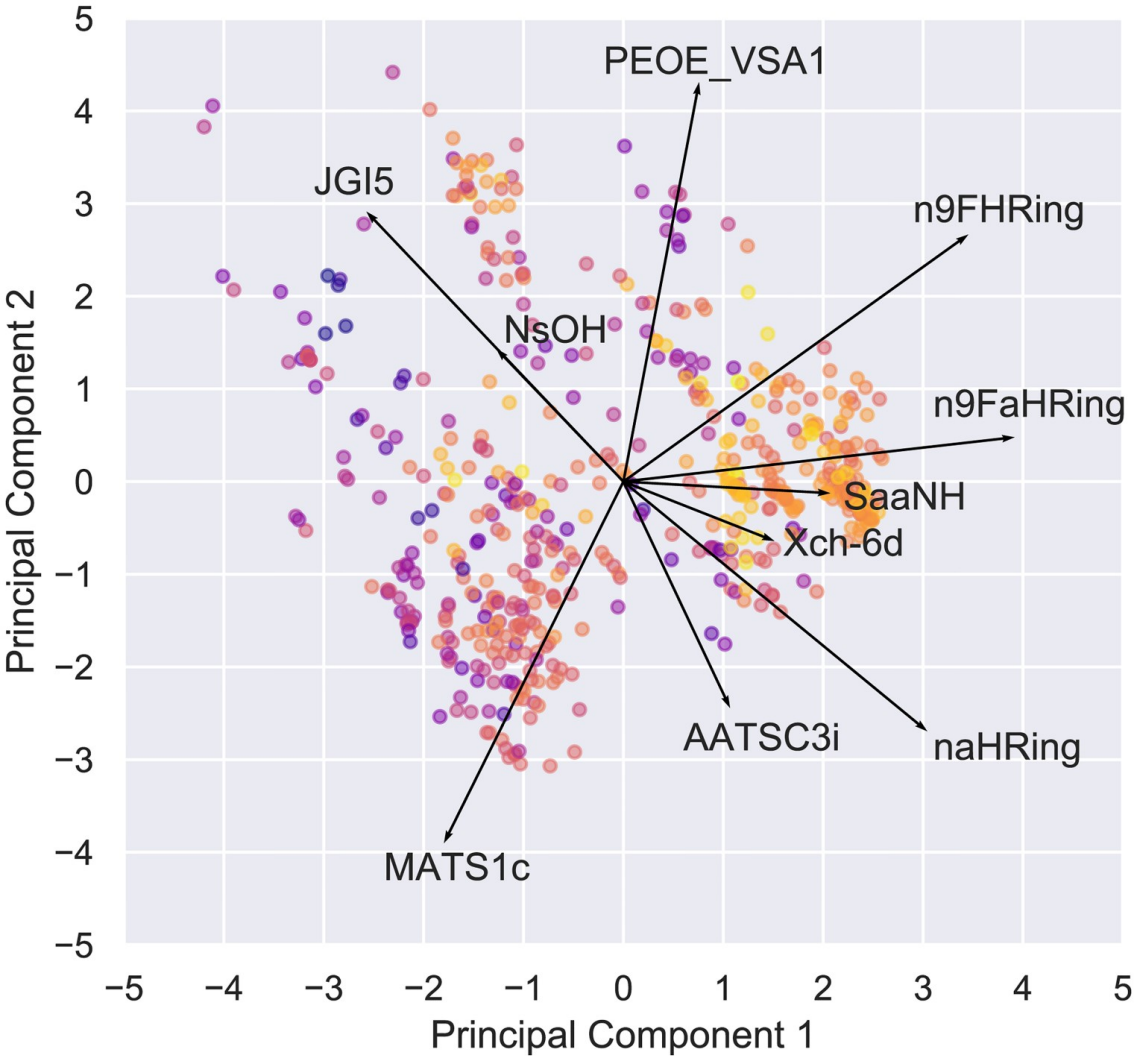
Biplot of the PCA analysis with all ten variables for all training molecules (loadings are out of scale). pIC_50_ is represented by color, where yellow are greater values and purple are lesser.

In order to visualize how different descriptors influence the variance of the data, a principal component analysis was conducted. Fig 10 shows the results obtained by the PCA analysis with all ten variables for all training molecules (the loading values of the descriptors are shown out of scale). The two first principal components represent 49.31% of the total variance. The activity values are represented by color, where the closer to yellow, the greater the activity, and the closer to purple, the smaller.

It is interesting to note that the variable that is the most important to the model, *naHRing*, is not well linearly correlated to the inhibitory effect (R^2^ = 22.4%), whereas less important variables are more correlated to the inhibition. However, *naHRing*, for example, does contribute to the variance in the data as much as other descriptors. This corroborates the non-linear regression abilities of the models that are not easily described by variance only.

### External validation

**Fig 11:**
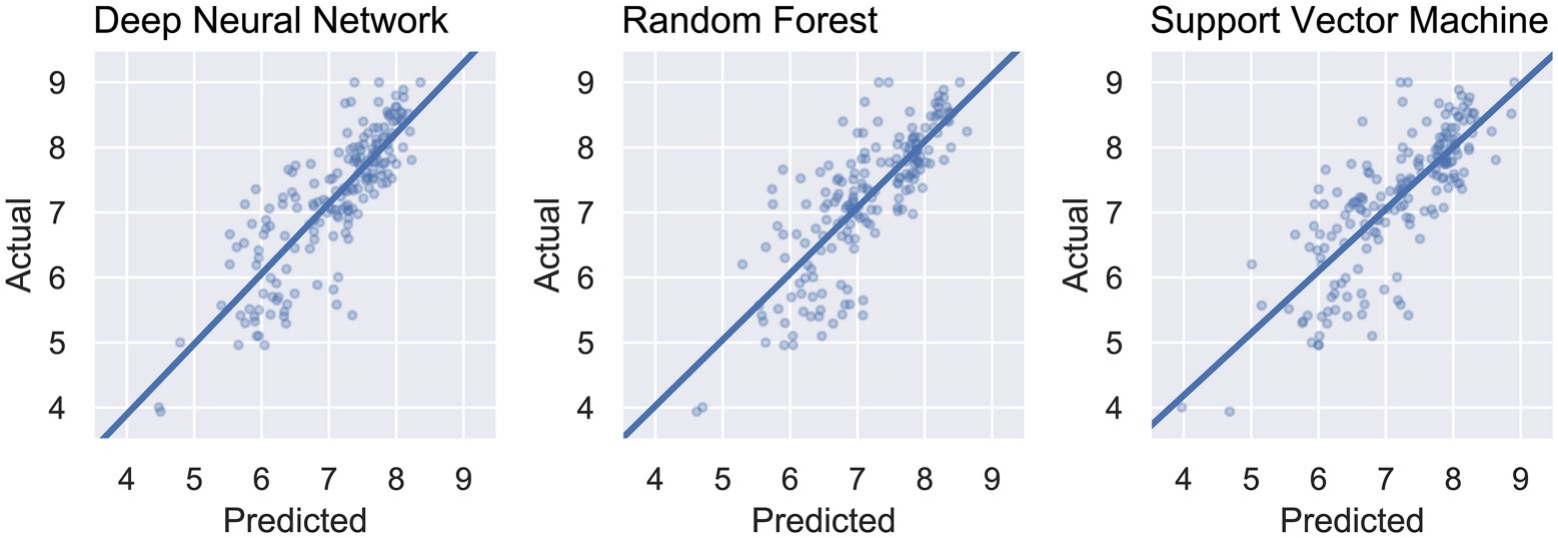
Actual and predicted pIC_50_ for the external validation from DNN, RF, and SVR models.

The results obtained by the external validation are displayed in Table 1. The dispersion plots for this validation step are presented in Fig 11. Again, the best performing model for all metrics was the neural network, which corresponds to the best generalization ability regarding the available data. As expected, the metrics for this validation are not as good as one from cross-validation, since the data presented to the predictors is completely independent of any data seen during training, feature selection or parameter tuning. The exception to this, however, is the SVR model that seems to perform better on the external data than on the cross-validation. This, however, may be due to chance, especially when considering the confidence interval for the error metrics. Even though DNN does perform better for all metrics, all models seem able to learn characteristics from the ligands and can predict inhibition data against ALK-5 fairly well.

## Conclusions

Cancer remains one of the highest-ranking causes of death around the world and, therefore, the identification of ALK-5 inhibitors has promising therapeutic importance. In this study, we developed machine learning models based on 2D molecular descriptors generated by the Mordred Python library software. The main objective of the resulting models was to predict the pIC_50_ (and consequently IC_50_) values for a significant number of compounds. The models were trained using a set of molecules gathered from ChEMBL. The main prediction model related to the ALK-5 inhibition was built by using a feedforward DNN model and the obtained results were compared to those obtained by two other ML methods (Random Forest and Support Vector Machines). It is relevant to point out that the DNN model demonstrated the best performance on both cross-validation and external validation experiments. The resulting model used ten 2D-molecular descriptors to predict the pIC_50_ values of the compound set (R^2^ = 0.658 on external validation). Moreover, this study demonstrated the abilities of the DNN models as important tools in drug discovery and design. The resulting model can also be further applied for virtual screening studies, a process in which libraries containing hundreds of thousands of compounds are rapidly searched as drug candidates with desired characteristics (in this case, inhibition of ALK-5) [49]. This minimizes the number of substances that end up being empirically tested and significantly speeds up the drug discovery and design. With the deep neural network model, it is possible to quickly infer the pIC_50_ value for any given single organic molecule with an expected mean absolute error of 0.480.
